# Quantitative and Qualitative Assessment of Fluorescence in Aesthetic Direct Restorations

**DOI:** 10.3390/ma15134619

**Published:** 2022-06-30

**Authors:** Zsuzsanna Bardocz-Veres, Melinda Székely, Pál Salamon, Előd Bala, Előd Bereczki, Bernadette Kerekes-Máthé

**Affiliations:** 1 Faculty of Dental Medicine, George Emil Palade University of Medicine, Pharmacy, Science, and Technology of Târgu Mureș, 38 Gh. Marinescu Str., 540139 Târgu Mureș, Romania; zsuzsanna.bardocz-veres@umfst.ro (Z.B.-V.); ballaelod59@gmail.com (E.B.); elodberecki@gmail.com (E.B.); bernadette.kerekes-mathe@umfst.ro (B.K.-M.); 2 Department of Bioengineering, Faculty of Economics, Socio-Human Sciences and Engineering, Sapientia Hungarian University of Transylvania [EMTE], Libertatii sq. 1, 530104 Miercurea Ciuc, Romania; salamonpal@uni.sapientia.ro; 3 Molecular Diagnostics Laboratory, Emergency County Hospital of Miercurea Ciuc, Dr. Denes Laszlo 2, 530173 Miercurea Ciuc, Romania

**Keywords:** dental materials, fluorescence properties, restorative materials, resin-based composite, enamel

## Abstract

Currently available direct restoration materials have been developed to have improved optical properties to interact with light in the same manner as the natural tooth. The objective of this study was to investigate the fluorescence of different enamel resin composites. In the present study, nine brands of enamel composites were tested in vitro, some of which are cited by manufacturers as having color adjustment potential. Fluorescence spectra of the composite specimens and the human natural enamel were measured with a fluorescence spectrophotometer immediately after preparation and after 6 months. Qualitative data of the specimens were also collected. Statistical analyses were conducted by Kruskal–Wallis and Mann–Whitney U nonparametric tests (*p* < 0.05). Almost all tested resin composites presented a significant decrease in the fluorescence values after a period of 6 months. There was no significant decrease in fluorescence in the case of Harmonize™ resin composite samples, which presented the lowest initial fluorescence values. The highest value in the reduction of the initial fluorescence intensity after 6 months (22.95%) was observed for the Charisma^®^ specimens. Composites with a color adjustment did not perform significantly better than other composites in terms of reduction in fluorescence intensity.

## 1. Introduction

Modern composites can reproduce the beauty of the appearance of a natural tooth, a potential reason for widespread use in the direct restorations of the anterior region. Currently available enamel direct composite materials differ not only in color but also in fundamental optical properties, such as translucency, opalescence, and fluorescence. These optical properties form the basis for clinical shade-matching. 

Light can be broken into three primary colors: red, green, and blue, and in their merging point, they produce three more colors, evidenced in prism light. Our brain perceives the reflected waves as color. The color that we perceive is the sum of all the colors reflected by the object. Color perception is regulated by absorption and reflection mechanisms. The human eye can perceive wavelengths included in a range between 380 and 760 nm. Light absorption and reflection phenomena work at the expense of the object’s translucency and opacity. Transparent and translucent objects allow light to pass partially or completely, while opaque objects block the passage of light [1].

The enamel is considered a crystalline tissue that, due to the arrangement of the prisms, translucency, and opalescence, confers the ability to transmit light to the underlying dentin, which features several nuances and three-dimensional aspects of color. The enamel is primarily responsible for regulating the tooth brightness and is characterized by a high degree of translucency and unique light effects. Enamel is composed mainly of hydroxyapatite and a lesser percentage of organic matter and water. The crystalline structure of the enamel prism allows light to pass with little restraint, while the organic interprismatic substance shows high opacity. The composition of the enamel enables a unique complex of reflection, transmission, and absorption of light. The interaction between the enamel and dentin makes the tooth an object that uniquely plays with light. Compared with other dental tissues, the enamel structure has a highly translucent appearance and a high degree of opacity. Even though no tooth structure is transparent, enamel allows light to pass through, providing translucent and opalescent effects [2,3].

**Figure 1:**
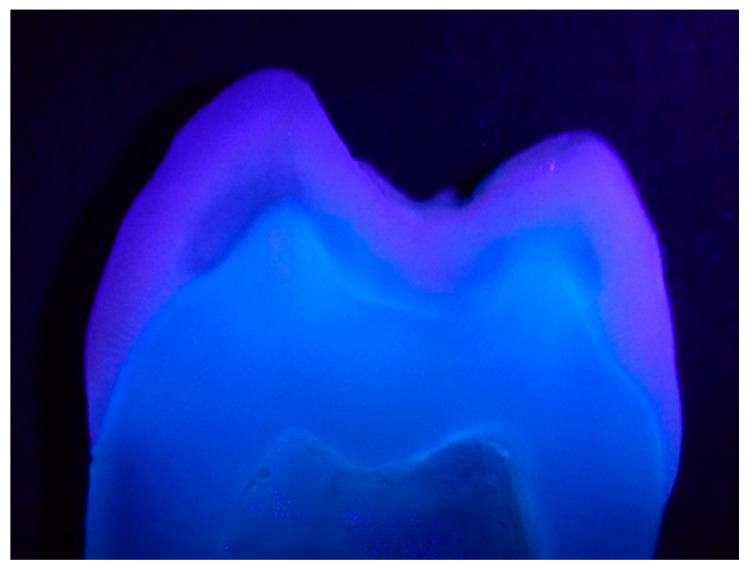
Fluorescence of a premolar slice (in 385 nm UV light). The digital equipment used was a DSLR camera (Nikon D3100, Nikon Corporation, Tokyo, Japan) equipped with a macro objective (Tamron 90 mm), ISO 200, f/22, exposure time: 1/200 s.

Fluorescence is defined as the optical property of a substance that, while exposed to the exciting irradiation, absorbs the light and consequently emits the light at a longer wavelength (Figure 1). Fluorescence, as an optical property, can determine the aesthetic quality, success, or failure of restorative treatment. Unfortunately, the fluorescence intensity of dental tissues and restorative materials cannot be certified visually, the phenomenon is evident under fluorescent light but still stands out significantly, although less obviously under natural light. The use of fluorescent materials has marked a revolution in aesthetic dentistry, therefore, today’s restorative materials that lack fluorescence are not considered to be optimal materials. The most important feature of the fluorescence is the light emission from inside, e.g., endodontic treatment and aging result in a decrease in the fluorescence because of protein loss, tissue mineralization, and pigmentation [4].

Fluorescence belongs to the family of photoluminescence processes, in which case, the molecules can emit light through electronically excited states. Photoluminescence is defined as the ability of bodies to emit certain types of light when subjected to invisible ultraviolet rays. It can be divided into two bodies: phosphorescent (bodies that have the ability to continue to emit visible light even after the removal of the ultraviolet rays) and fluorescents (bodies that emit visible light only during exposure to ultraviolet rays).

The first studies related to fluorescence in natural teeth defined that the teeth presented white–blue fluorescent properties when exposed to low-intensity radiation of the ultraviolet rays. This characteristic makes the natural teeth whiter and brighter in daylight, giving them an aspect of vitality and naturalness.

Fluorescence is a phenomenon capable of absorbing light energy of ultraviolet origin and re-emitting it in the visible light spectrum in the form of blue–violet light. This means that the absorption of electromagnetic waves invisible to the human eye is converted by the body irradiated with ultraviolet light, which re-emits it as visible energy [5].

Restorative materials that stimulate the natural tooth color have different particle size distribution and optical characteristics: they absorb some rays while transmitting and reflecting others. The synergy among them creates the colors perceived by the eye. Composite resins and dental ceramics are materials that absorb a relatively large amount of light [6]. 

**Figure 2:**
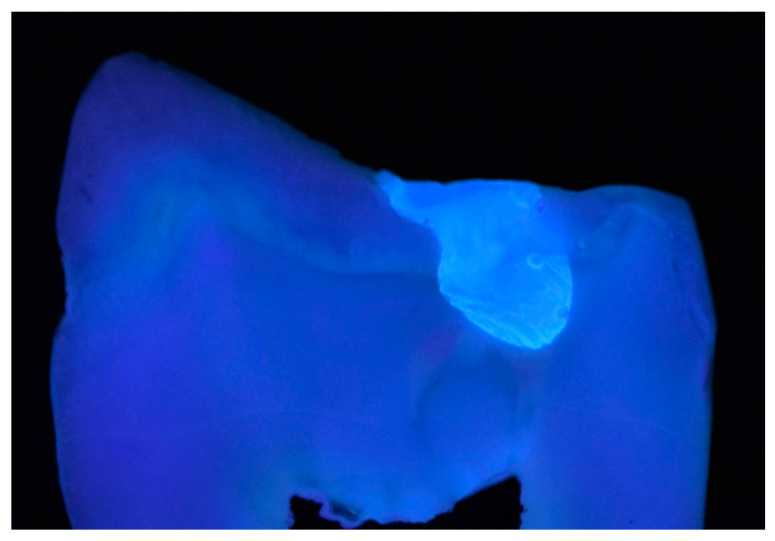
Image of a cross-section premolar slice in UV light (385 nm), revealing the differences in fluorescence between dental tissues and a restoration with Charisma^®^. The digital equipment used was a DSLR camera (Nikon D3100) equipped with a macro objective (Tamron 90 mm), ISO 200, f/22, exposure time: 1/200 s.

The enamel and dentin interrelation in the natural tooth determines their color through the processes of reflection and refraction of light. This means that restorative materials need to have similar optical properties to the dental structure, making the restorations almost undetectable. The dentin and the enamel differ in fluorescence (Figure 2).

Manufacturers have included special agents from metals like europium, terbium, ytterbium, and cerium to reproduce the phenomena of fluorescence. Clinically, fluorescence contributes to the aspect of the vitality of the restoration and helps to obtain the correct luminosity. Different composites have different degrees of fluorescence, depending on the manufacturer’s approach or the optical properties of the material. The lighter the chroma, the more fluorescent the material becomes. However, the fluorescence of the composites still does not completely mimic that of natural teeth [7,8].

Several studies have dealt with the opalescence and fluorescence properties of resin composites. Since UV light causes fluorescent emission in dental resin composites, this may influence the opalescence property and translucency of materials. Therefore, inclusion or exclusion of the UV component of illumination may have an influence on the translucency and masking effect [9].

The objective of the present study was to evaluate, in vitro, the fluorescence intensity of resin composites, focusing on the direct restoration of the enamel. The null hypotheses tested were that the fluorescence intensity of composite samples: (i) does not differ significantly from the enamel samples and (ii) does not reduce over time.

## 2. Materials and Methods

A total number of 9 different brands of restorative materials used for the direct restoration of enamel were analyzed. The materials included in the study are presented in Table 1. 

In the first part of the study, six specimens from each of the nine brands of the composite were prepared according to the manufacturers’ instructions. The sample size was calculated using a sample size calculator (SSCALC, at a confidence level of 95%, the value of the confidence interval was 10). Discs of 5 mm in diameter and 2 mm in thickness were made using a silicone mold (Elite HD Putty Soft, Zhermack, SpA, Badia Polesine (RO), Italy ). A total number of 54 specimens were prepared. The resin was inserted in a single increment for each specimen, followed by the positioning of a polyester sheet and a glass plate on the surface. A force of 20 N was applied to eliminate the excess material. The curing of the specimens was realized using a hand light-curing unit (Noblesse Wireless LED, Max Dental Co, Seokcheon-ro, Ojeong-gu, Bucheon-si, Gyeonggi-do, Korea) for 20 s from one side of the mold, with an intensity setting of 1000 mW/cm^2^. As a control group, we prepared eight natural enamel slices of 5 mm × 2 mm each from upper premolars extracted for orthodontic reasons. Enamel specimens were obtained by slicing the tooth with a high-speed diamond disc under water cooling. The dimension of the specimens was verified using a digital micrometer caliper (Powerfix, OWIM, Neckarsulm, Germany). As a polishing protocol, 600 grit sandpaper was applied for every specimen. The specimens were stored in plastic containers with artificial saliva to avoid desiccation which could bias the measured parameters. Just before the measurements, specimens were removed from the containers and blot-dried. 

For the quantitative measurements, a fluorescence spectrophotometer was used (Cary Eclipse, Fluorescence Spectrophotometer, Agilent Technologies, Santa Clara, CA, USA). The samples were introduced in Quartz Microplate (Hellma Analytics, material: Quartz Glass, wells diameter: 6.6 mm, dimensions: 14.5 mm × 127 mm × 85.5 mm). A fluorescence emission spectrum was recorded at an excitation wavelength of 395 nm; the emission measurement range was 400–600 nm. The data were analyzed by the SpectraGryph 1.2 spectroscopy software. To assess the fluorescence intensity changes over time of the composite resin specimens, the same measurements were repeated after 6 months.

In the second part of the study, we collected qualitative data on the materials’ fluorescence. Disc specimens of 12 mm in diameter and 1 mm in thickness were made using a custom-made silicone mold. Light curing was performed with the same protocol as described above. After curing, specimens were removed from the mold. Two specimens were made for each brand (a total number of 18 specimens) and all were made by the same operator (EB) at the same room temperature and humidity.

The qualitative evaluation of the fluorescence intensity was assessed by a qualitative visual method [10] by two of the authors (ZSBV and BKM). Therefore, the samples were introduced in a custom-made black box and illuminated by a UV light source at the intensity of 385 nm. All the specimens and the natural enamel samples were photographed using a DSLR camera (Nikon D3100) with a macro lens (Tamron 90 mm Macro lens) from the same distance and standardized adjustments (ISO 200, f/22, exposure time: 1/200 s). For the analysis, a blind-type experiment was utilized, in which the evaluator was unaware of the trademark of the composite resin that was being evaluated. The order of disposition of the specimens was predetermined. Each evaluator received a form to be filled in with the responses they observed and they were instructed to evaluate the degree of fluorescence by a numerical value: 0 = low fluorescence, 1 = medium fluorescence, 2 = high fluorescence.

The collected data were statistically analyzed using Microsoft Excel spreadsheets, Kruskal-Wallis and Mann-Whitney U nonparametric tests (GraphPadPrism), using *p* values < 0.05.

## 3. Results

**Figure 3:**
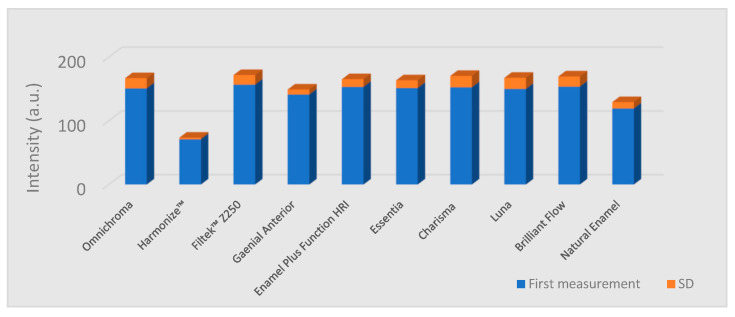
Column graph of the fluorescence intensity values (in arbitrary units) from the first measurement, representing the mean and standard deviation (SD) of each type of composite and the enamel.

**Figure 4:**
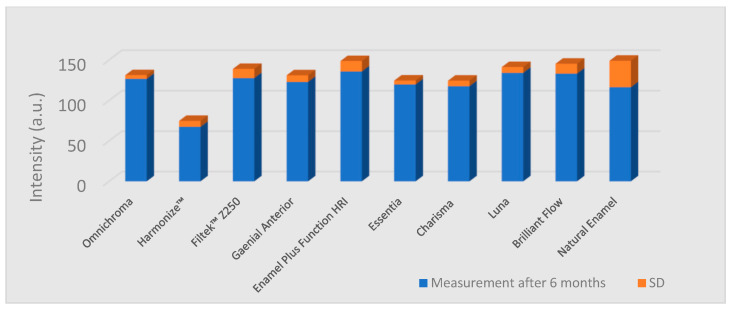
Column graph of the fluorescence intensity values (in arbitrary units) measured after 6 months, representing the mean and standard deviation (SD) of each type of composite and the enamel.

The lowest fluorescence intensity values were measured in the case of Harmonize™ (Kerr Dental) composite specimens (Table 2). The measured values in this group were significantly lower than the values of the enamel samples group (*p* = 0.005) and all the other composite groups (*p* = 0.01). All the other composite groups showed significantly higher values than natural enamel (*p* = 0.01). Fluorescence intensity data are given in Figure 3 and Figure 4. The reduction of the values after 6 months and statistical significance are shown in Table 2.

**Figure 5:**
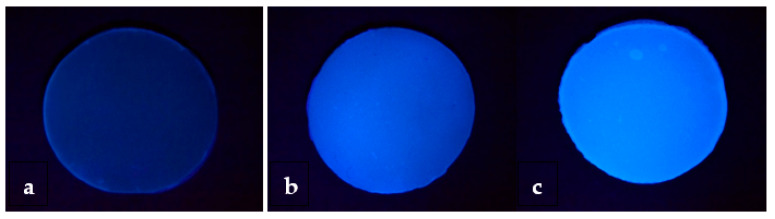
Samples under an ultraviolet light source showing the different fluorescence levels of the materials. Samples with different values: (**a**) low fluorescence (value 0); (**b**) medium fluorescence (value 1); (**c**) high fluorescence (value 2).

The qualitative assessment showed that only one of the composite materials belongs to the low fluorescence group, another composite belongs to the medium fluorescence, including all the brands with special shades (Table 3; Figure 5). 

## 4. Discussion

Although we found multiple studies analyzing the fluorescence properties of the dental materials, no previous publication focused just on the enamel resin composites. The fluorescence of a resin composite is essential to reproduce the optical properties of dental hard tissues. Not all manufacturers consider this aspect important. Besides this, the precise composition of these materials is proprietary information, therefore, it is particularly hard to define which component is responsible for the fluorescent effect. In the international scientific literature, we found only comparative studies between different material brands or studies that compare natural teeth with restorative materials [4,5,10,11].

Garrido et al. reported increased fluorescence intensity as a function of time in the case of Filtek Z350 XT (3M ESPE). They considered that light absorption increases with aging, which leads to a slight increase in fluorescence emission over time, and this could be related to the organic components of this resin. In our study Filtek Z250, from the same manufacturer, showed a decrease in the fluorescence intensity after 6 months. A possible explanation could be the degradation of the organic complexes over time and with respect to the fact that rare earth metals have a low light absorption [12]. Although, in the present study, the fluorescence of Filtek Z250 was higher than that of other composites because Z250 has a smaller content of light scattering particles. These differences can be due to the nano behavior of Z250 and the presence of special particles in their composition. The difference in fluorescence of the composites tested in our study seems to be attributed to the difference in their composition and size of fillers.

All studies showed that composite resins that present fluorescent properties comparable to the enamels’ have a mixture of several agents in their composition to achieve this characteristic. The organic and inorganic components, the type and the particle size of these, and any irregularity of the particles, can decrease light absorption and reflection. Resin composite has a high degree of translucency and value, absorbing, dispersing, and reflecting light in a similar way to dental structures. To understand that the final fluorescence is not equal to the sum of the fluorescence of each luminescent agent used in the material, manufacturers include fluorescent additives such as europium, cerium, and ytterbium, to improve the aesthetics of composite resins under all lighting conditions [13].

Previous studies have reported a gradual loss of the fluorescence properties of resin composites after aging, have included small sample sizes. With little differences between the brands, in all the nine resin composite specimens, a reduction in the fluorescence intensity was observed, however statistically significant differences could not be revealed in all cases. The fluorescence intensity of the specimens dropped to approximately 15% of the initial values after 6 months. Therefore, more awareness of the fluorescence properties of resin-based composites is needed [14].

Meller et al. examined composite samples of different brands and types, and they concluded that the fluorescence of different shades of the same brand is variable. They reported descriptive results and showed the different maximum intensity of fluorescence, which indicates the absence of standard fluorescent properties among different shades, even from the same brand [15].

The fluorescence of material reaches its optimal level at a certain concentration due to the interaction of light and fluorescent particles. Basically, after exceeding the optimal threshold, the fluorosed light is absorbed by other particles and decreases the efficiency of fluorescence (quenching effect). This phenomenon occurs when the thickness increases. In our study, the density of fluorescent particles in 1 mm thickness was too low to show the quenching effect. Thus, although thickness affects the fluorescence, this effect has an ascending trend to some extent and then descends due to the quenching effect [16].

Based on the fact that to generate distinct shades of composites, manufacturers add different amounts of chemicals like pigments, initiators, inhibitors, and activators, which influence the fluorescence phenomenon, Conceição et al. presented in their study a simple method to determine differences in fluorescence and reflectance. The Fluorescence and Reflectance Scale allow the examiner to identify a specific brand or restrict the possibilities down to two brands. This information about the fluorescence value of a dental restoration could help forensic experts in cases of identification, especially when antemortem data is limited [17].

Considering that each manufacturer uses specific compounds and combine these chemicals in different proportions, the fluorescence and reflectance phenomenon is unique for each manufacturer or brand.

In their study, Park et al. incorporated an organic fluorescent agent in varying concentrations in a resin matrix and measured the fluorescence intensity. Other manufacturers tried rare earth hybrid ions to polymerize into the resin matrix and as agents to achieve fluorescent composites [18].

Jablonski et al. showed a total reduction of 54.2% of the initial fluorescence intensity of Charisma^®^, however, the present study revealed a reduction of 22.95% of the initial fluorescence intensity after a half year by Jablonski et al. (23.5%). The differences between the study by Jablonski et al. and the present study probably result from the fact that in their study, two-color shades were evaluated, and in this study, only shade A2 was evaluated. We need to mention the fact that the specimens in their study were exposed to extreme conditions to simulate the aging process, while in our study, no aging conditions were used [19].

Stoleriu et al. analyzed the fluorescence properties of two composite resins; both materials presented low fluorescence, the results being in accordance with the present study. We would like to point out that in their study, both dentin and enamel shades were evaluated, and the results showed a higher emission of the fluorescence in dentine shades compared to the enamel shades [20].

Researchers conceptualized dental fluorescence around a wavelength between 430–450 nm, and they showed that surface characteristics of test specimens could cause changes in the optical properties of materials, either by the form of storage or even by the time taken for analysis [9]. Other studies showed that the different forms of storage of test specimens and different polishing protocols do not cause significant changes in the intensity of the fluorescence of the test specimens [11]. In the present study, specimens were stored in physiological serum, as we considered them more stable than artificial saliva.

The human eye is exposed to the range of light that forms the electromagnetic spectrum, which can decompose at various wavelengths, but only a small spectrum, the visible light spectrum initiates the process of color perception. However, the light source has numerous emission forms that differ in wavelength, so the perception of colors can suffer changes according to the amount of light that falls upon the object [21,22]. This could be one of the possible variables in the present study. Visual analysis of the images obtained under UV light showed extreme differences in the fluorescent contrast of the specimens, however, most of the specimens showed high fluorescence intensity. The evaluation of the data obtained was made by relating the results of the statistical analysis and the qualitative interpretation of the images obtained under UV light. In the case of Harmonize composite samples, very low initial fluorescence intensity values were assessed. Thus, the decrease in this sample group was not significant, while other materials, which had high initial values, were showing significant decreases. 

## 5. Conclusions

Enamel composite resins’ fluorescence intensity may differ significantly from the natural enamel’s fluorescence intensity, presenting significantly lower or higher values than the natural enamel. Within the limitations of the present study, we can state that all the tested resin composites presented a decrease in fluorescence values after 6 months. The null hypotheses of the present study were rejected.

The results could be used as a reference value in the development of aesthetic restorative enamel composites. Although the first experimental data are encouraging, it would be recommended to carry out a controlled study with a larger number of shades, more brands of resin composites, and furthermore, in vivo studies, to verify possible changes in fluorescence of the materials in the oral environment. 

## Figures and Tables

**Table 1:** Materials included in the study.

Materials	Composition	Manufacturer	Shade
Omnichroma	UDMA, TEGDMA, uniform-sized supra-nano spherical filler (260 nm spherical SiO_2_-ZrO_2_), composite filler (SiO_2_-ZrO_2_)	Tokoyama Dental, Tokyo, Japan	One shade (Special)
Harmonize™	Bis-GMA, Bis-EMA, TEGDMA, spherical silica, and zirconia particles 5 to 400 nm formed from a molecular suspension in ART, barium glass	Kerr Dental, Orange, CA, USA	A2
Filtek™ Z250	Bis-GMA, UDMA, and Bis-EMA; 66% of filler: Zirconium/Silica	3M ESPE Dental Products, St. Paul, MN, USA	A2
Gaenial Anterior	UDMA, dimethacrylate co-monomers. Filler: silica, strontium, lanthanoid fluoride (16–17 µm), silica (>100 nm) fumed silica	GC Corporation, Tokyo, Japan	A2
Enamel Plus Function HRI	Bis-GMA, UDMA, butanediol dimethacrylate Nano-hybrid composite content of filler (80% weight)	Micerium, Avegno, Italy	EF3 (Special)
Essentia	UDMA, Bis-MEPP, Bis-EMA, Bis-GMA, TEGDMA, Filler: pre polymerized fillers, barium glass, fumed silica	GC Corporation, Tokyo, Japan	LE (Special)
Charisma	Bis-GMA, TEGMA, Ba-Al-F glass fillers, pre-polymerized filler, pyrogenic silica, initiator	Heraeus Kulzer, Hannau, Germany	A2
Luna	UDMA/Bis-EMA/TEGDMA, (61%) SAS, AS0.02-2 μm, 200–400 nm	SDI GmbH, Cologne, Germany	A2
Brilliant Flow	Bis-GMA, Bis-EMA, TEGDMA, barium glass, silanized silica (0.6 µm), 42%vol	Coltene-Whaledent, Altstatten, Switzerland	A2/B2

**Table 2:** Reduction of the fluorescence intensity values after the second measurement for each material and the enamel specimens; *p* values are based on Mann–Whitney nonparametric tests.

Materials	Reduction after 6 Months, in Percentage	*p*-Value
Omnichroma	16.08%	0.01 *
Harmonize™	5.22%	*0.149*
Filtek™ Z250	18.66%	0.01 *
Gaenial Anterior	13.05%	0.01 *
Enamel Plus Function HRI	11.39%	0.01 *
Essentia	20.91%	0.006 *
Charisma	22.95%	0.01 *
Luna	10.64%	*0.07*
Brilliant Flow	13.33%	0.03 *
Natural Enamel	2.48%	*0.16*

* Significant differences.

**Table 3:** The final values provided by the evaluators according to the defined fluorescence groups.

Specimen	Dominant Fluorescence Group
Harmonize™	0
Gaenial Anterior	1
Filtek™ Z250	2
Omnichroma	2
Enamel Plus Function HRI	2
Essentia	2
Charisma	2
Luna	2
Brilliant Flow	2

## Data Availability

Data supporting results can be found at the first author.
